# Primary Care Provider HIV PrEP Knowledge, Attitudes, and Prescribing Habits: A Cross-Sectional Survey of Late Adopters in Rural and Suburban Practice

**DOI:** 10.1177/21501319221147254

**Published:** 2023-01-10

**Authors:** Jarrett Sell, Rensa Chen, Christopher Huber, Jessica Parascando, Jonathan Nunez

**Affiliations:** 1 Penn State Health Hershey Medical Center, Hershey, PA, USA; 2 Yale School of Medicine, New Haven, CT, USA

**Keywords:** HIV, prep, primary care, rural, suburban

## Abstract

**Objective:**

Pre-exposure prophylaxis (PrEP) is a recommended strategy for HIV prevention, yet PrEP prescribing rates in primary care remain low. The aim of this study was to further describe the current knowledge, attitudes, and prescribing behaviors of HIV PrEP in primary care providers with a focus on the perceived barriers and facilitators to PrEP prescribing.

**Methods:**

Cross-sectional survey of primary care providers at rural and suburban practices in a large academic institution.

**Results:**

Survey response rate was 48.0% (n = 134). Most respondents (96.3%) reported little clinical experience in care of persons living with HIV. Respondents self-reported positive attitudes and high overall knowledge of PrEP with low prescribing rates and less comfort with lab testing. More respondents are asked about PrEP by patients (54%) than start conversations about PrEP with patients (39%). Family Physicians and providers 5 to 10 years from completion of training overall reported higher knowledge, attitudes and prescribing behaviors. Lack of PrEP education was identified as the greatest barrier and an electronic medical record order set as the greatest facilitator to prescribing PrEP.

**Conclusions:**

With the goal to end the HIV epidemic, PrEP provision in nonurban primary care settings may be an important strategy for increased access to PrEP and reduced HIV transmission. This study, which includes a variety of providers that possess high knowledge, yet low experience prescribing PrEP, likely demonstrates the limitations of interventions which solely focus on provider education. System-based practice solutions, such as order sets, may be needed to target infrequent prescribers of PrEP.

## Introduction

HIV continues to be a global and national health issue, with an estimated 1.2 million people in the United States living with HIV and an annual rate of 38 000 new infections per year.^1^ While the U.S. Food and Drug Administration approved oral tenofovir and emtricitabine in 2012 for pre-exposure prophylaxis (PrEP) to reduce the risk of HIV acquisition, the Centers for Disease Control and Prevention (CDC) estimate that of the 1.2 million persons who could benefit from PrEP, only 25% had been prescribed PrEP in 2020.^2^ Even though PrEP prescribing has been increasing over time, the COVID-19 pandemic was estimated to reduce PrEP prescriptions by 22% in a modeling study that looked at national pharmacy data from January 2017 through March 2021.^3^ Access to PrEP remains a priority for reducing the rate of new infections and ending the HIV/AIDS epidemic, and the United States Preventive Services Taskforce (USPSTF) gave PrEP an “A” grade in 2019, recommending that clinicians offer PrEP to persons who are at high risk of HIV acquisition.^4^ Access to PrEP is particularly a concern in non-urban settings in which studies have shown lower HIV testing rates and later stage initial HIV diagnosis.^5^ PrEP awareness and adoption has been particularly poor nationally in people who inject drugs, who face additional barriers to PrEP access in rural settings.^6,7^ While general awareness and comfort in prescribing PrEP by primary care providers has increased over time,^8^ there remains a minority that actually prescribe PrEP.^9^

Previous studies have identified that primary care providers’ likelihood of prescribing HIV PrEP is based on training, attitudes toward PrEP effectiveness, perceptions of patient risk behaviors, stigma, and provider purview, in which primary care providers perceive PrEP to be better prescribed by specialists.^10-13^ Early adopters of PrEP are more likely to be infectious disease specialists or primary care providers who also manage HIV, have high PrEP knowledge, perceive PrEP as safe, and believe that PrEP is less likely to increase risk behaviors. Provider practice-related characteristics, such as years in practice, frequency of taking a detailed sexual history, perceived HIV risk of patients, and frequency of screening and management of sexually transmitted infections (STIs) and HIV have also been shown to influence PrEP prescribing.^10,12,14-16^ Lastly, provider knowledge of patient eligibility, cost, insurance coverage, lab testing, and the risks and benefits of PrEP have been identified as barriers to PrEP prescribing.^17,18^ Despite these identified barriers, there are limited studies exploring facilitators to PrEP prescribing by primary care providers.

The aim of this study was to describe the current knowledge, attitudes, and prescribing behaviors of HIV pre-exposure prophylaxis in primary care providers with a focus on the perceived barriers and facilitators to PrEP prescribing.

## Materials and Methods

### Study Design

A cross-sectional survey study was conducted to assess primary care providers’ prescribing of HIV PrEP. The primary outcomes were PrEP knowledge, attitudes, barriers, facilitators, and self-reported prescribing behaviors, including number of prescriptions prescribed within the past 6 months.

### Participants

All primary care providers, including faculty, advance practice providers, and residents, in the Family and Community Medicine and Internal Medicine departments of a large academic institution were recruited for this study (n = 279). Providers practice in over 16 unique suburban and rural practice settings in central Pennsylvania and were included in the study if they provided direct outpatient primary care. HIV prevalence, incidence and PrEP prescribing rates in the practicing counties of the study population are provided in Table 1 and compared with state average rates.

**Table 1.** HIV Prevalence, Incidence and PrEP Prescribing Rates in Practicing Central PA Counties of the Study Population, Compared with State Average Rates in 2019^*^.

	#PLWH/100 000	% compared to state average	New HIV Dx/100 000	% compared to state average	PrEP use/100 000	% compared to state average
PA state average	334		9		123	
Dauphin county	511	152.99	10.3	114.44	120	97.56
Lebanon county	179	53.59	5.9	65.56	55	44.72
Lancaster county	175	52.40	5.5	61.11	60	48.78
Cumberland county	135	40.42	4.2	46.67	50	40.65
Center county	144	43.11	Not reported	Not reported	81	65.85

Abbreviations: PLWH, people living with HIV per 100 000 population; Dx, diagnoses; PA, Pennsylvania.

* AIDSVu. Local Data: Pennsylvania. https://aidsvu.org/local-data/united-states/northeast/pennsylvania/

### Survey

An anonymous survey was adapted from the previously published PCP PrEP Survey,^12^ and was distributed in January of 2021 (Supplemental File 1). The voluntary survey, which featured 40 Likert scale questions, was hosted in REDCap (Research Electronic Data Capture).^19^ Demographic data was collected in regard to self-reported gender, sexual orientation, race, and ethnicity as data has shown that there are gender, racial, and ethnic differences in PrEP access, as studies have shown that provider demographics may be important in patient choice of providers.

### Data Analysis

Quantitative analysis was performed using the R statistical program (version 3.5.2) to generate descriptive statistical analyses and tables. A chi-squared or Fisher’s exact test was used for categorical data and a Wilcoxon rank sum or Kruskal-Wallis rank sum test was used for continuous data.

This study (#16543) was determined to be not human subjects research and exempt from Institutional Review Board review on November 19th, 2020.

## Results

A total of 134 primary care providers completed the survey for a response rate of 48.0%. Table 2 shows that respondents include similar numbers of men and women, with the majority of respondents self-reporting as heterosexual (92.5%), White (71.7%), and non-Hispanic (98.4%). Half (50.7%) of respondents were within 5 years of training, with a greater number working in Family Medicine (69.5%) and as faculty (66.4%). Most respondents (96.3%) report little clinical experience in HIV management and care for 5 or less patients living with HIV.

**Table 2.** Participant Self-Reported Demographics (n = 134).

Demographics	Overall sample(n = 134)
Age, mean (SD)	37.8 (12.0)
Gender, n (%)	
Male	71 (53.0)
Female	60 (44.8)
Transgender or gender non-conforming	0 (0)
Chose not to answer	3 (2.2)
Sexual orientation, n (%)	
Heterosexual	124 (92.5)
Lesbian, gay, or bisexual	5 (3.7)
Other	1 (0.7)
Chose not to answer	4 (3.1)
Race, n (%)	
White	96 (71.7)
Black or African American	3 (2.2)
Asian or Asian American	23 (17.2)
More than one race/other	5 (3.7)
Chose not to answer	7 (5.2)
Ethnicity, n (%)	
Non-Hispanic	127 (98.4)
Hispanic	2 (1.6)
Chose not to answer	5 (3.8)
Years in practice after training, n (%)	
<5 years	68 (50.7)
5-10 years	23 (17.2)
>10 years	43 (32.1)
Specialty, n (%)	
Family medicine	91 (67.9)
Internal medicine	40 (29.9)
Chose not to answer	3 (2.2)
Clinic regional campus affiliation, n (%)	
Hershey, Pennsylvania	106 (79.0)
State College, Pennsylvania	26 (19.4)
Chose not to answer	2 (1.6)
Role, n (%)	
Faculty	89 (66.4)
Resident	44 (32.8)
Chose not to answer	1 (0.8)
Degree, n (%)	
MD/DO	112 (83.6)
PA/CRNP	22 (16.4)
Prescribe antiretroviral therapy, n (%)	11 (8.2)
Number of patients with HIV, n (%)	
0	62 (46.3)
1-5	67 (50.0)
6-25	5 (3.7)

Table 3 indicates that respondents self-reported high overall knowledge of PrEP and screening for STIs, while reporting lower knowledge of PrEP side effects, baseline lab testing, and ongoing lab safety monitoring. Attitudinal responses demonstrate that respondents consider PrEP to be effective, safe, and unlikely to change sexual risk-taking practices. Over half (54.3%) of respondents have been asked about PrEP by a patient, but only 39% of providers have initiated a conversation about PrEP with a patient. While close to half (46%) have previously prescribed PrEP and 64.1% report being moderately/extremely comfortable prescribing PrEP, a minority (43.8%) reported that they were likely to prescribe PrEP in the next 6 months.

**Table 3.** Primary care providers’ self-reported HIV pre-exposure prophylaxis (PrEP) knowledge, attitudes, and prescribing behaviors (n = 128).

	Overall sample(n = 128)*n*, (%)
Knowledge^*^	Good/Very Good/Excellent
Before today, how would you rate your knowledge of PrEP?	71 (55.5)
Before today, how would you rate your knowledge of PrEP’s potential side effects (eg, renal dysfunction)?	53 (41.4)
Before today, how would you rate your knowledge of baseline lab testing recommended by the Centers for Disease Control and Prevention (CDC) before starting PrEP?	49 (38.3)
Before today, how would you rate your knowledge of ongoing lab safety monitoring recommended by the Centers for Disease Control and Prevention (CDC) for continued prescribing of PrEP?	48 (37.6)
Before today, how would you rate your knowledge of screening for sexually transmitted infections (STIs) other than HIV recommended by the Centers for Disease Control and Prevention (CDC) for patients prescribed PrEP?	81 (63.4)
Attitudes^†^	Moderately/Extremely
How effective do you think PrEP is in preventing acquisition of HIV among people who take it every day as prescribed?	126 (98.5)
Based on your understanding of PrEP side effects, how safe is PrEP?	120 (93.7)
How likely are you to prescribe PrEP in the next 6 months?	56 (43.8)
If you identified a patient at high risk for HIV acquisition, how comfortable would you be with prescribing PrEP?	82 (64.1)
How likely do you think the patient would be to increase his/her sexual risk-taking practices (eg, decrease condom use) as a result of being on PrEP?	30 (23.4)
How likely do you think the patient would be to decrease his/her sexual risk-taking practices (eg, increase condom use) as a result of being on PrEP?	22 (17.2)
Prescribing behaviors	
Have you ever been asked about PrEP by a patient? (yes) n, (%)	69 (54)
Have you ever initiated a conversation about PrEP with a patient? (yes) n, (%)	50 (39)
Have you ever prescribed PrEP to a patient? (yes) n, (%)	59 (46)
How many prescriptions for PrEP have you prescribed in the past 6 months? Mean (SD)	1.4 (0.5)

* Knowledge questions on a 1 to 5 Likert scale (1 = “poor”; 5 = “excellent”).

† Attitude questions on a 1 to 4 Likert scale (1 = “not at all”; 4 = “extremely”).

Providers in Table 4 rank “lack of PrEP training and education” as the largest barrier to prescribing PrEP (2.8 out of 4), with “lack of clinic guidelines and protocols and costs” as the second largest barrier (both 2.5 out of 4). Respondents most highly indicated that an order set in the electronic medical record (EMR) that details recommended testing would most facilitate PrEP prescribing (3.5 out of 4). Clinical pharmacy support (3.4 out of 4), peer support (3.3 out of 4), and access to guidelines and protocols (3.2 out of 4) were also ranked highly as potential facilitators for PrEP prescribing.

**Table 4.** Primary Care Providers’ Perceived Barriers and Facilitators to HIV Pre-Exposure Prophylaxis (PrEP) Prescribing (n = 128).

	Average score (SD)
Barriers	
Lack of provider training/education regarding PrEP	2.8 (1.1)
Lack of clinic guidelines/protocol for prescribing/monitoring PrEP	2.5 (1.0)
Lack of insurance coverage and out-of-pocket patient costs for PrEP and related care (eg, lab work)	2.5 (0.9)
Clinical and lab monitoring requirements (eg, seeing patient and obtaining HIV tests and STI screening every 3 months; checking renal function every 6 months)	2.1 (0.9)
Staffing/time constraints related to risk reduction and PrEP adherence counseling (also medication knowledge/counseling, adverse effects, etc.)	2.1 (0.9)
Facilitators	
Electronic Medical Records (EMR) Order Set that details recommended testing	3.5 (0.7)
Clinical pharmacist support (tracking adherence and assisting with medication counseling)	3.4 (0.8)
Peers who are knowledgeable about or supportive of PrEP provision within your practice	3.3 (0.8)
Access to resources such as PrEP prescription guidelines and protocols	3.2 (0.9)

Abbreviations: STI, sexually transmitted infection.

All responses on a 1 to 4 Likert scale (“1 = not at all likely to be a barrier/facilitator”; “4 = extremely likely to be a barrier/facilitator”)

In Table 5, which compares sub-groups of respondents, Family Medicine providers report, with statistical significance, being more likely to have been asked about PrEP by a patient, being comfortable prescribing PrEP to patients at high risk for HIV acquisition, having ever prescribed PrEP, and most likely to prescribe PrEP in the next 6 months, than Internal Medicine providers. However, the average number of PrEP prescriptions prescribed in the past 6 months by FM (1.4) and IM (1.2) providers was similar and low, but statistically different. When comparing groups based on the number of years after training, those providers that were 5 to 10 years from training overall reported higher knowledge, attitudes, than those with more or less post-training clinical experience. Additionally, providers 5 to 10 years after completion of training had a trend toward higher average number of PrEP prescriptions prescribed in the past 6 months (1.7) in comparison to those less than 5 years after training (1.3) and over 10 years from training (1.4), although the difference did not reach statistical significance (*P* < .05).

**Table 5.** Comparison Subgroup Analysis of Differences in HIV Pre-Exposure Prophylaxis (PrEP) Knowledge, Attitudes and Prescribing Behaviors Based on Specialty and Years after Completion of Training.

	Family medicine (n = 91)	Internal medicine (n = 40)	*P*-value	<5 years after training (n = 68)	5-10 years after training (n = 23)	>10 years after training (n = 43)	*P*-value
Before today, how would you rate your knowledge of PrEP? Mean (SD)	2.7 (1.0)	2.5 (1.0)	.16	2.4 (0.9)	3.3 (0.9)	2.8 (0.9)	<.001^*^
Before today, how would you rate your knowledge of PrEP’s potential side effects (eg, renal dysfunction)? Mean (SD)	2.4 (1.2)	2.2 (1.0)	.22	2.1 (1.1)	3.0 (1.1)	2.4 (1.0)	.005^*^
Before today, how would you rate your knowledge of baseline lab testing recommended by the Centers for Disease Control and Prevention (CDC) before starting PrEP? Mean (SD)	2.5 (1.4)	2.1 (1.1)	.15	2.1 (1.2)	3.1 (1.3)	2.5 (1.2)	.004^*^
Before today, how would you rate your knowledge of ongoing lab safety monitoring recommended by the Centers for Disease Control and Prevention (CDC) for continued prescribing of PrEP? Mean (SD)	2.5 (1.3)	2.1 (1.1)	.14	2.0 (1.2)	3.1 (1.3)	2.5 (1.2)	.001^*^
Before today, how would you rate your knowledge of screening for sexually transmitted infections (STIs) other than HIV recommended by the Centers for Disease Control and Prevention (CDC) for patients prescribed PrEP? Mean (SD)	3.0 (1.2)	2.7 (1.1)	.19	2.5 (1.2)	3.5 (1.0)	3.2 (1.1)	<.001^*^
How effective do you think PrEP is in preventing acquisition of HIV among people who take it every day as prescribed? Mean (SD)	3.7 (0.5)	3.7 (0.5)	.88	3.6 (0.5)	3.8 (0.4)	3.8 (0.5)	.08
Based on your understanding of PrEP side effects, how safe is PrEP? Mean (SD)	3.3 (0.6)	3.2 (0.6)	.18	3.1 (0.6)	3.6 (0.5)	3.4 (0.5)	.002^*^
How likely are you to prescribe PrEP in the next 6 months? Mean (SD)	2.6 (1.1)	2.1 (0.8)	.02^*^	2.3 (1.1)	2.9 (1.0)	2.4 (0.9)	.08
If you identified a patient at high risk for HIV acquisition, how comfortable would you be with prescribing PrEP? Mean (SD)	2.9 (1.1)	2.5 (0.9)	.03^*^	2.4 (1.0)	3.3 (0.9)	3.1 (0.9)	<.001^*^
How likely do you think the patient would be to increase his/her sexual risk-taking practices (eg, decrease condom use) as a result of being on PrEP? Mean (SD)	1.9 (0.8)	2.0 (0.8)	.52	1.9 (0.9)	1.8 (0.6)	2.0 (0.9)	.41
How likely do you think the patient would be to decrease his/her sexual risk-taking practices (eg, increase condom use) as a result of being on PrEP? Mean (SD)	1.8 (0.7)	1.8 (0.8)	.75	1.8 (0.8)	1.7 (0.6)	1.9 (0.8)	.67
Have you ever been asked about PrEP by a patient? (yes) n, (%)	59 (64.8)	10 (25)	<.001^*^	26 (38.2)	17 (73.9)	26 (60.4)	.001^*^
Have you ever initiated a conversation about PrEP with a patient? (yes) n, (%)	38 (41.8)	12 (30)	.24	20 (29.4)	12 (52.2)	18 (41.9)	.061
Have you ever prescribed PrEP to a patient? (yes) n, (%)	47 (51.6)	12 (30)	.03^*^	21 (30.9)	17 (73.9)	21 (48.8)	<.001^*^
How many prescriptions for PrEP have you prescribed in the past 6 months? Mean (SD)	1.4 (0.5)	1.2 (0.4)	.003^*^	1.3 (0.5)	1.7 (0.7)	1.4 (0.5)	.48^*^

* *P*-value for between group comparison using chi-squared or Fisher’s exact test for categorical data and a Wilcoxon rank sum or Kruskal-Wallis rank sum test for continuous data with significance <.05.

## Conclusions

Our study adds to the literature by describing HIV PrEP knowledge, attitudes and prescribing practices of a variety of primary care providers in rural and suburban practice settings and highlights the current gap in broader prescribing of PrEP. While the adoption of new drugs is often faster in specialists,^20,21^ Edelman et al^22^ demonstrated that a majority of general internists favored integrating PrEP into primary care, and our respondents reported positive attitudes regarding the safety and efficacy of PrEP. According to the diffusion of innovations theory,^23,24^ which is a framework for looking at the factors that influence the adoption of new technologies (ie, prescribing a new medication), respondents in our study were predominantly late adopters, prescribing PrEP with low frequency, and providing little direct care for those living with HIV. The focus of providers primarily practicing in non-urban settings adds to the literature, which has shown reduced PrEP access and training in rural family medicine residency programs^25^ and in rural areas.^26-28^ Our study showed that a majority of providers have been asked by patients about PrEP, but that providers initiate conversations about PrEP less frequently, which is consistent with a chart review in the Veterans Administration, which found that patients initiated the majority of conversations about PrEP.^29^

Respondents identified education as the highest barrier, which is consistent with other studies. Importantly, cost was identified as a significant perceived barrier, although the Affordable Care Act necessitates insurance coverage of PrEP medication, associated lab testing, and clinical services,^30^ which may represent a lack of provider awareness of PrEP provision policies and experience in prescribing PrEP as well as delays in insurance compliance with all aspects of PrEP provision. While gaps in coverage for PrEP exist, particularly in those that are under or uninsured,^31^ the federal government reiterated in July 2021 that PrEP services should be covered by insurance companies without cost-sharing.^32^ Provider hesitancy to prescribe PrEP due to cost concerns contrasts with many other preventative health services that are routinely recommended by providers, who often are familiar with discussing costs of other preventative services they routinely order.

With the goal of ending the national HIV epidemic, PrEP provision in nonurban primary care settings may be an important strategy for increased access to PrEP and reduced HIV transmission. In the counties included in this study, PrEP prescribing rates, when compared to state averages, were lower than the relative incidence when compared to state averages in all counties with reported data (Table 1), demonstrating gaps in PrEP prescribing in these regions. This study, which includes a variety of providers with high knowledge, yet low experience prescribing PrEP, likely demonstrates the limitations of interventions which solely focus on provider education. While older studies, which focused on specialist providers with HIV management experience, found significant differences in self-reported knowledge and attitudes regarding safety and efficacy that correlated with PrEP prescribing,^10,33^ our study found overall high self-reported knowledge and positive attitudes toward PrEP prescribing, which suggests that additional work to reduce systems level barriers may be now needed to improve PrEP access in primary care.

Limitations of this study include a small cohort at a single regional academic health care center and may not be generalizable to other settings. As the study was conducted at the start of the COVID-19 pandemic, it is possible that reduced in-person office visits during the early pandemic could reduce provider reporting of prescribing behaviors, although this likely would not significantly affect provider attitudes and knowledge. Respondents also could be more likely to respond positively to PrEP due to social desirability bias, which was minimized by the anonymous nature of the survey.

Our study uniquely identified an electronic medical record order set as the number one facilitator for PrEP prescribing by respondents, which may indicate the need to develop system-based practice solutions targeted at infrequent prescribers of PrEP, which is consistent with a systemic review of barriers to PrEP prescribing and found the need to better optimize PrEP delivery.^13^ While evidence-based policy statements from the USPSTF and CDC have been clear about the benefits of PrEP, primary care providers do not have sufficient time with patients to address all the current preventative health recommendations,^34^ making it crucial to look at processes that streamline PrEP initiation and maintenance. Avery et al found that implementing an electronic medical record-based reminder effectively increased HIV screening among primary care patients, while education and practice feedback alone did not.^35^ Studies have identified gaps in PrEP lab testing, finding HIV testing is not ordered 25% of the time before initiation of PrEP prescribing, with lower rates of follow-up testing as recommended by the CDC.^11,36^ Future research is needed to further describe late adopters to PrEP prescribing and determine if systems-based interventions, such as EMR order sets and protocols, can both increase access and appropriate lab monitoring for PrEP in primary care.

## Supplemental Material

sj-docx-1-jpc-10.1177_21501319221147254 – Supplemental material for Primary Care Provider HIV PrEP Knowledge, Attitudes, and Prescribing Habits: A Cross-Sectional Survey of Late Adopters in Rural and Suburban Practice Click here for additional data file. Supplemental material, sj-docx-1-jpc-10.1177_21501319221147254 for Primary Care Provider HIV PrEP Knowledge, Attitudes, and Prescribing Habits: A Cross-Sectional Survey of Late Adopters in Rural and Suburban Practice by Jarrett Sell, Rensa Chen, Christopher Huber, Jessica Parascando and Jonathan Nunez in Journal of Primary Care & Community Health
