# Supplementary material for: Primary Care Provider HIV PrEP Knowledge, Attitudes, and Prescribing Habits: A Cross-Sectional Survey of Late Adopters in Rural and Suburban Practice
Source: J Prim Care Community Health. 2023 Jan 10;14:21501319221147254. doi: 10.1177/21501319221147254 (PMC9834790; doi:10.1177/21501319221147254)
Supplement: sj-docx-1-jpc-10.1177_21501319221147254 – Supplemental material for Primary Care Provider HIV PrEP Knowledge, Attitudes, and Prescribing Habits: A Cross-Sectional Survey of Late Adopters in Rural and Suburban Practice [file sj-docx-1-jpc-10.1177_21501319221147254.docx]

**Penn State Health Primary Care PrEP Survey**

**Initial Survey**

The purpose of this study is to learn about primary care providers' knowledge, attitudes, and behavior regarding pre-exposure prophylaxis ("PrEP") for HIV prevention. This anonymous survey should take 10 - 15 minutes to complete.

Your participation is completely voluntary and you do not have to participate if you do not want to. You may skip some questions you do not feel comfortable answering.

| **Are you a primary care provider?** | - Yes - No |
| --- | --- |
| **What is your specialty?** | - Internal Medicine - Family and Community Medicine |
| **Do you currently provide direct clinical care in the outpatient setting?** | - Yes - No |
| **Where is your primary clinic based?** | - Hershey - State College |
| **Please select your role:** | - Faculty - Resident |
| **Please select your year in residency (*if resident*)** | - PGY1 - PGY2 - PGY3 |
| **Please select your degree:** | - MD - DO - PA - CRNP - Other: ________ |

PrEP stands for “Pre-Exposure Prophylaxis”, the use of any medicine to prevent a disease before exposure to that disease. For the purposes of this survey, the term refers to the use of an oral antiretroviral medication taken on a daily basis by people at high risk of exposure to HIV to prevent HIV infection.

Truvada, a combination of two antiretroviral medications tenofovir and emtricitabine, was approved for PrEP in 2012 by the FDA and recommended in 2014 by the CDC. The FDA has also approved Descovy for PrEP to reduce the risk of HIV infection through sex, excluding those who have receptive vaginal sex.

| **Before today, had you heard of PrEP?** | - Yes - No |
| --- | --- |

**For the following questions, please select your level of knowledge as:**

Excellent: expert-level, up-to-date in research

Very Good: proficient, not up-to-date in research

Good: recognize there are side effects, but may not be as up-to-date and would likely review prior to prescribing

Fair: general knowledge, need to review

Poor: heard of it

|  | **Poor**  **1** | **Fair**  **2** | **Good**  **3** | **Very Good**  **4** | **Excellent**  **5** |
| --- | --- | --- | --- | --- | --- |
| **Before today, how would you rate your knowledge of PrEP?** | ○ | ○ | ○ | ○ | ○ |
| **Before today, how would you rate your knowledge of PrEP's potential side effects (e.g., renal dysfunction)?** | ○ | ○ | ○ | ○ | ○ |
| **Before today, how would you rate your knowledge of baseline lab testing recommended by the Centers for Disease Control and Prevention (CDC) before starting PrEP?** | ○ | ○ | ○ | ○ | ○ |
| **Before today, how would you rate your knowledge of ongoing lab safety monitoring recommended by the Centers for Disease Control and Prevention (CDC) for continued prescribing of PrEP?** | ○ | ○ | ○ | ○ | ○ |
| **Before today, how would you rate your knowledge of screening for sexually transmitted infections (STIs) other than HIV recommended by the Centers for Disease Control and Prevention (CDC) for patients prescribed PrEP?** | ○ | ○ | ○ | ○ | ○ |

**The following set of questions asks about your opinions based on what you know about PrEP. It's OK if you're not very familiar with PrEP - just answer to the best of your ability.**

|  | **Not at all**  **1** | **Slightly**  **2** | **Moderately**  **3** | **Extremely**  **4** |
| --- | --- | --- | --- | --- |
| **How effective do you think PrEP is in preventing acquisition of HIV among people who take it every day as prescribed?** | ○ | ○ | ○ | ○ |
| **Based on your understanding of PrEP side effects, how safe is PrEP?** | ○ | ○ | ○ | ○ |
| **How likely are you to prescribe PrEP in the next 6 months?** | ○ | ○ | ○ | ○ |
| **If you identified a patient at high risk for HIV acquisition, how comfortable would you be with prescribing PrEP?** | ○ | ○ | ○ | ○ |
| **How likely do you think the patient would be to increase his/her sexual risk-taking practices (e.g., decrease condom use) as a result of being on PrEP?** | ○ | ○ | ○ | ○ |
| **How likely do you think the patient would be to decrease his/her sexual risk-taking practices (e.g., increase condom use) as a result of being on PrEP?** | ○ | ○ | ○ | ○ |

| **Have you ever been asked about PrEP by a patient?** | - Yes - No |
| --- | --- |
| **Have you ever initiated a conversation about PrEP with a patient?** | - Yes - No |
| **Have you ever prescribed PrEP to a patient?** | - Yes - No |
| **How many prescriptions for PrEP have you prescribed in the past 6 months?** | - Zero - 1-5 - 6-25 - >25 |

**Rate the degree to which each of the following is a potential barrier to prescribing PrEP at your primary clinic:**

|  | **Not at all likely to be a barrier**  **1** | **2** | **3** | **Extremely likely to be a barrier**  **4** |
| --- | --- | --- | --- | --- |
| **Lack of provider training/education regarding PrEP** | ○ | ○ | ○ | ○ |
| **Lack of clinic guidelines/protocol for prescribing/monitoring PrEP** | ○ | ○ | ○ | ○ |
| **Clinical and lab monitoring requirements (e.g., seeing patient and obtaining HIV tests and STI screening every 3 months; checking renal function every 6 months)** | ○ | ○ | ○ | ○ |
| **Staffing/time constraints related to risk reduction and PrEP adherence counseling (also medication knowledge/counseling, adverse effects, etc.)** | ○ | ○ | ○ | ○ |
| **Lack of insurance coverage and out-of-pocket patient costs for PrEP and related care (e.g., lab work)** | ○ | ○ | ○ | ○ |

**Rate the degree to which each of the following would facilitate your prescribing PrEP at your primary clinic:**

|  | **Not at all likely to be a facilitator**  **1** | **2** | **3** | **Extremely likely to be a facilitator**  **4** |
| --- | --- | --- | --- | --- |
| **Access to resources such as PrEP prescription guidelines and protocols** | ○ | ○ | ○ | ○ |
| **Electronic Medical Records (EMR) Order Set that details recommended testing** | ○ | ○ | ○ | ○ |
| **Clinical pharmacist support (tracking adherence and assisting with medication counseling)** | ○ | ○ | ○ | ○ |
| **Peers who are knowledgeable about or supportive of PrEP provision within your practice** | ○ | ○ | ○ | ○ |

| **What percent of your time do you spend in direct patient care?** | - DROP DOWN (0-100%) - Prefer not to share - Not applicable |
| --- | --- |
| **Of the patients you currently provide primary care for at your clinic/office, approximately how many patients are HIV-positive?** | - Zero - 1-5 - 6-25 - >25 |
| **Do you prescribe antiretroviral therapy for chronic management of HIV?** | - Yes - No |
| **With respect to gender, how do you self-identify?** | - Female - Male - Transgender - Gender non-conforming - Choose not to answer |
| **With respect to sexual orientation, how do you self-identify?** | - Heterosexual - Gay - Lesbian - Bisexual - Other (please specify): _______ - Choose not to answer |
| **I have been in practice:** | - Less than 5 years - 5 to 10 years - 11 to 15 years - 16 to 20 years - Greater than 20 years |
| **What is your age?** | __________ |
| **With respect to race, how do you self-identify? (Check all that apply)** | - White - Black or African American - American Indian and Alaska Native - Asian or Asian American - Native Hawaiian and Other Pacific Islander - Other (please specify): _______ - Choose not to answer |
| **Do you self-identify as being Hispanic/Latino?** | - Yes - No |
